# Tailored Multifaceted Strategy for Implementing Fundamental Evidence-Based Nursing Care: An Evaluation Study

**DOI:** 10.3390/nursrep14040297

**Published:** 2024-12-18

**Authors:** Signe Eekholm, Karin Samuelson, Gerd Ahlström, Tove Lindhardt

**Affiliations:** 1 Department of Health Sciences, Faculty of Medicine, Lund University, Sölvegatan 19, P.O. Box 117, SE-221 00 Lund, Sweden; karin.samuelson@med.lu.se (K.S.); gerd.ahlstrom@med.lu.se (G.A.); 2 Department of Internal Medicine, Copenhagen University Hospital Herlev and Gentofte, Gentofte Hospitalsvej 2, 2nd. Floor, DK-2900 Hellerup, Denmark; tove.lindhardt.damsgaard@regionh.dk

**Keywords:** behavioural change, evaluation, evidence-based practice, feasibility, implementation outcomes, implementation strategy, nursing care

## Abstract

**Background/Objectives**: Extensive research has emphasised the persistent challenges and failures in providing hospitalised patients with fundamental evidence-based nursing care, often resulting in grave consequences for patient safety. Recommendations from implementation research indicate that a tailored theory- and research-based implementation strategy targeting contextual determinants can optimise the implementation of evidence-based clinical practice for the benefit of patients. This study evaluated the feasibility of an implementation strategy designed to improve the quality of nursing care by targeting behavioural and environmental barriers in a hospital setting. **Methods**: Proctor’s conceptual model for implementation was applied to evaluate the strategy based on eight outcomes: adoption, acceptability, appropriateness, fidelity, feasibility, penetration, sustainability, and costs. Data collection methods included field observations, informal and focus group interviews, registrations, and audits of electronic patient records. **Results**: The strategy was adoptive, acceptable, appropriate, and feasible in targeting complex environmental and behavioural determinants (at the individual, team, and management level), enabling successful implementation of fundamental evidence-based nursing care. However, fidelity, feasibility, and sustainability were challenged by competing organisational demands and time constraints. **Conclusions**: The tailored, multifaceted strategy proved effective in addressing complex environmental and behavioural determinants across multiple levels, facilitating the implementation of fundamental evidence-based nursing care in a clinical practice. Further testing and larger-scale studies is needed to assess the strategy’s transferability and its impact on nursing-sensitive patient outcomes in different clinical settings.

## 1. Introduction

Systematic reviews and cross-sectional studies continually report a risk to patient safety among hospitalised patients due to inadequate and insufficient quality of nursing care [1,2,3,4]. The current literature provides mounting evidence that nursing activities oriented on meeting patient fundamental needs, i.e., basic human needs such as nutrition, mobility, communication, hygiene, physical comfort, and emotional support, are often inadequately addressed or absent [2,4,5]. Though, there is a strong scientific consensus that fulfilling fundamental patient needs is essential for high-quality, safe care [4,5,6,7]. Furthermore, fundamental patient needs including physical, psychological, and relational needs must be met according to the best available evidence and tailored to individual patient preferences to ensure effective and high-quality treatment and care [5,8,9,10]. According to Braithwaite et al. [11], only 60% of nursing care provided is consistent with evidence-based recommendations; also, 30% of performed care is wasted or of low value (i.e., ineffective, inappropriate, or poorly cost-effective care), and 10% of delivered care is harmful [11]. Moreover, a plethora of research studies indicates that inadequate nursing care affects 55–98% of hospitalised patients, with frail elderly patients with complex needs and multimorbidity being at high risk [1,2,12,13]. Inadequate nursing care is acknowledged as a strong predictor for adverse advents such as complications, prolonged hospitalisations, readmissions, and, most notably, mortality [3,9,11]. This highlights the critical need for changes in clinical practice in favour of improving the quality of nursing care and the promotion of delivering fundamental evidence- based nursing care.

A number of national and international initiatives have been attempted to emphasise the importance of nursing care through political initiatives, strategies, the optimisation of nursing education, the retention of registered nurses (RNs), and increased hiring of support staff [4,14,15,16]. These initiatives aim to support RNs in delivering fundamental, evidence-based nursing care that meets individual patient needs. However, they have shown limited effect [4,14,15,16,17]. Problematically, the majority of change initiatives have not been developed to target change at multiple levels as recommended in implementation science [18,19], which is particularly relevant in healthcare settings, where healthcare professionals collaborate across disciplines and organisational borders. The focus lies mostly on individual or organisational determinants only.

Considering that nursing care delivery is often hindered not only by individual barriers but also by time constraints, heavy workloads, lack of management support, insufficient interdisciplinary collaboration, and organisational hierarchy, it is essential to develop multi-level change initiatives to promote nursing practice and evidence uptake in care initiatives [18]. Implementation science offers a promising solution to this pressing issue, advocating for the design of tailored, theory- and research-based multifaceted strategies to achieve the desired goal [19,20,21,22]. Accordingly, the first step towards successful change is a thorough identification of both hindering and facilitating determinants within the specific context where change is intended. Furthermore, it is strongly recommended that change initiatives are tailored to the context and grounded in theory or research, as such approaches have been shown to facilitate successful implementation and clinical practice change, ultimately enhancing patient safety [21,22]. Although these recommendations are promising, we still face challenges in achieving consistent success, underscoring the need for further studies and the refinement of strategies to enhance nursing practice and patient safety [4,14,15,16]. Acknowledging this major challenge, we previously conducted an ethnographic study [23] aimed at identifying the determinants that hinder or facilitate the delivery of fundamental, evidence-based nursing care in a hospital setting. Findings from that study revealed four primary barriers. Firstly, at the individual level, RNs focused on treatment-related tasks, driven by the biomedical model, which led to the neglect of patients’ fundamental care needs. Additionally, the majority of RNs demonstrated insufficient knowledge, unclear role perceptions, and challenges in prioritising, planning, delivering, and documenting fundamental nursing care. Secondly, at the interdisciplinary level, consistent interruptions, delegation of non-nursing tasks to RNs, and unannounced visits from other professionals disrupted care delivery. Thirdly, at the environmental level, poor coordination with external organisations (e.g., when the central kitchen does not deliver enough food for patients despite RNs orders, requiring RNs to retrieve additional food themselves) limited the time RNs had to provide nursing care. Lastly, inadequate management support was identified as crucial for fostering a culture of evidence-based nursing care [23]. Based on these determinants, we designed a tailored, multifaceted theory- and research-based implementation strategy, as well as a structured implementation plan to promote nursing practice and eliminate barriers at multiple levels that hinder the delivery of fundamental evidence-based nursing care [24]. The aim was to evaluate the multifaceted implementation strategy and its implementation process. To our knowledge, this is the first study to provide scientific and practical guidance for eliminating comprehensive contextual and behavioural barriers that limit the delivery of fundamental evidence-based nursing care in clinical practice. We believe that this study will offer research-based insights and recommendations for implementation efforts that can help healthcare providers optimise clinical practice, benefiting both patients and healthcare professionals, particularly RNs.

## 2. Materials and Methods

### 2.1. Design and Theoretical Framework

This study applied the process evaluation approach [25] to determine if, how, and why the multifaceted implementation strategy was successful, or not, utilising the implementation process outcomes defined by Proctor’s implementation outcomes framework [26]. The implementation outcomes included acceptability, adoption, appropriateness, feasibility, cost, fidelity, penetration, and sustainability, which were measured using multiple data collection methods (Table 1).

### 2.2. Setting and Participants

The study was conducted over a period from June 2020 to July 2021 in a medical unit for respiratory diseases (25 beds). The unit is located in the Department of Internal Medicine at a large university hospital in the Capital Region of Denmark, providing treatment and care for patients with pulmonary diseases, for example, pneumonia and pulmonary fibrosis. The hospital provides specialist healthcare to approximately 174,000 patients annually.

All healthcare professionals employed in the unit—RNs (*n* = 20), licenced practical nurses (*n* = 8), physicians (*n* = 5), dieticians (*n* = 1), and physiotherapists (*n* = 2)—were invited to participate in this study. The unit nurse manager, two assistant nurse managers, and the clinical nurse specialist, with a master’s degree, were responsible for executing the implementation strategy in cooperation with the researchers *(n* = 2, first and last author). Among the admitted patients, 195 patients were consecutively selected by purposive sampling to evaluate the implementation interventions involving patient care.

### 2.3. Implementation Strategy

The strategy was developed based on findings from a prior study, identifying that fundamental nursing care interventions such as nutritional support, fluid therapy, oral care, and ambulation were sporadically, or sometimes never, delivered in alignment with evidence-based recommendations for admitted patients [27]. An ethnographic study [23] (described in a particular background) informed us about determinants hindering or facilitating delivery of fundamental evidence-based nursing care. Findings from this study were used to develop a multifaceted theory-and research-based implementation strategy (described in detail elsewhere [24]), utilising the intervention mapping framework’s six steps approach [28]. The intervention mapping’s six steps consisted of the following: (1) developing a logic model of the problem, (2) developing a logic model of change by defining change objectives, (3) designing and selecting theory-based implementation interventions, (4) planning the interventions through a co-design approach, (5) developing a structured plan for implementation, and (6) developing an evaluation plan. The end product of this approach was a strategy targeting environmental determinants and behaviours (e.g., attention, knowledge, skills, social influence) at the individual, team, and management level that, together, aimed to promote implementation of fundamental evidence-based nursing care.

An overview of the implementation strategy is presented in Table 2. The strategy further included a structured implementation plan that described in detail who needed to do what, when, and how, as well as a comprehensive monitoring plan for data collection. During the detailed planning of the structured implementation plan, it was crucial to plan the execution of the interventions during existing work schedule (e.g., conducting lectures during the unit’s weekly lecture time) to minimise implementation overload and mitigate time constraints.

### 2.4. Project Organisation

The steering group—which comprised two researchers (the first and last authors), a head nurse, and a nurse manager—facilitated the adoption of the implementation process. The first author served as the primary investigator and was responsible for data collection and supporting the strategy execution in the medical unit. A designated group, referred to hereafter as key persons, comprised the nurse manager, two assistant nurse managers, the clinical nurse specialist, and three RNs with expertise in nursing documentation, nutrition, and respiratory treatment were responsible for executing the implementation interventions. All key persons were individually trained by the first author in executing each of the implementation interventions, such as reminders, facilitation, elimination, feedback, and bedside training. Each training session lasted 45 min, while the bedside training comprised three sessions, each lasting five hours. The key persons and the researcher (first author) met once a week for 30 min to evaluate the implementation process and plan any necessary adjustments of the implementation interventions. This process involved utilising findings from the weekly observations and audits, as well as incorporating the experiences of key persons during their execution of the strategy.

### 2.5. Data Collection

To evaluate the implementation process and understand the impact of the strategy and its interventions, we collected data according to a data collection plan, based on Proctor’s implementation outcomes framework, during the eight-month intervention period and at the three- and six-month follow-ups [26]. This framework included the following eight outcomes [26] (Table 1), often perceived to be the ‘gold standard’ in implementation research: adoption, acceptability, appropriateness, fidelity, feasibility, penetration, sustainability, and costs. Data were collected through (1) participatory field observations, including informal interviews (individual and in groups); (2) focus group interviews; (3) registration of the frequency of implementation strategy activities; and (4) audits of electronic patient records. Findings from audits and observations were evaluated and discussed at weekly information meetings (Table 2) with the key persons. The aim was to adjust the execution of the implementation interventions, if needed. Furthermore, findings were strategically used to provide individual feedback (Table 2) to the registered nurses on personal achievements by the key persons or the researcher and provide motivation among study participants. An overview of the implementation outcomes and data collection methods is presented in Table 1.

#### 2.5.1. Participatory Field Observations and Informal Interviews

The participatory field observations, which included informal interviews [29], followed a semi-structured observation guide and were carried out one to three times a week. Observations were directed at key persons who executed the implementation interventions and RNs who carried out their practice in cooperation with the interdisciplinary team—including physicians, physiotherapists, dieticians, and licenced practical nurses—and external services (kitchen staff). Informal interviews were conducted individually or in groups during observations in order to clarify any uncertainties encountered during the observations. Field notes were written in a logbook during observations and interviews; these notes included information regarding the date, context, participants, communication among study participants, and personal reflections of what occurred during the observations or interviews. Overall, there were 69 periods of observations, which ranged between 0.5 h and 8 h.

#### 2.5.2. Focus Group Interviews

Focus group interviews (*n* = 8, 3–8 persons in each group) were conducted with the key persons and staff (each profession individually) following a semi-structured interview guide based on the implementation outcomes framework [26]. Focus group interviews were conducted to explore the staff’s and key persons’ perspectives on and experiences with the implementation interventions. The interview guide included questions such as, Can you elaborate on how you find implementation interventions to support your provision of fundamental evidence-based nursing care? The interviews lasted approximately 30 min–45 min and were digitally recorded and transcribed verbatim.

#### 2.5.3. Registration

The frequency and execution of implementation interventions were registered (e.g., how often and how many RNs had received bedside training), which included the costs of the development of materials and time spent on the project by the key persons.

#### 2.5.4. Audits

Audits on electronic patient records were conducted once a week (for patients admitted for ≥24 h) to assess the impact of the strategy on delivering nursing care interventions regarding patients’ individual needs. Audits were carried out for a total of 22 weeks and at three- and six-months follow-up. However, after eighteen weeks of the intervention period, audits had to be put on hold for 10 weeks due to the COVID-19 pandemic. Data were collected following a data collection guide structured according to the evidence-based guidelines criteria for nursing care interventions: nutritional support, fluid therapy, oral care, ambulation, oxygen therapy, and sputum mobilisation (Supplementary Materials Table S1). Additionally, we examined variables that included the registration of nursing care plans and whether these plans incorporated evidence-based clinical guideline recommendations and patient involvement. To identify if the relevant and sufficient nursing care interventions had been initiated in accordance with the patient’s individual needs, the experienced clinical nurse specialist in cooperation with the first author assessed admitted patients’ individual needs for nursing care interventions regarding the patient’s clinical status and diagnosis. Thereafter, the proportion of patients who had received the nursing care interventions was identified through documented delivery in electronic patient records. With regard to the audit, all admitted patients who were able to provide consent were interviewed to assess whether they were involved, guided, and informed regarding the individual plan for nursing care (e.g., oral care and fluids therapy). Overall, 195 audits and patient interviews were conducted.

#### 2.5.5. Follow-Up

Audits, field observations, and focus group interviews were repeated after the intervention period at three and six months to assess the ‘penetration’ and ‘sustainability’ of the strategy.

### 2.6. Analysis

Qualitative data from the focus group interviews, field observations, and informal interviews were integrated into one text body and included information of the data source and date. All data were analysed using deductive content analysis [30,31]. Two researchers read through the data to gain an understanding. The first author coded data, condensed the content, and categorised data according to Proctor’s outcomes [26]. The last author critically reviewed the categories against the raw data and discussed it in depth with the first author to reach a consensus. The result was critically questioned and discussed with the second and third authors. For the quantitative data, electronic patient records and registration sheets were analysed using descriptive statistics and the SPSS version 25 software.

## 3. Results

### 3.1. Adoption

The key persons had an overall positive attitude and willingness to adopt the multifaceted implementation strategy and felt comfortable executing the implementation interventions. This was fostered by previous experience and the training sessions. Weekly evaluation meetings, facilitated by data from weekly monitoring, made them aware of the importance of their efforts, which also increased their motivation for continuing the implementation process. Furthermore, they realised the relevance of adopting specific implementation interventions, for example, bedside training.

*“This shows that the RNs we employ must immediately receive bedside training. Assessment of RNs’ competences alone is not enough. They need to receive bedside training right away at their employment. They must know how to perform fundamental and also evidence-based nursing care”.*

(Informal interview with a key person, ID29.)

### 3.2. Acceptability

In general, RNs expressed a positive attitude towards the part of the implementation strategy that primarily targeted behaviour change among the RNs.

*“I can see great potential in this process and knowing that we will be part of research… There is a professional pride in it for me, and it means a lot”.*

(Informal interview with an RN, ID25.)

There was acceptance and willingness among other healthcare professionals to be a part of the implementation process, as it was experienced as meaningful.

*“It’s like water on my mill, because everything you’ve just presented is something I’ve been asking for in years”.*

(Informal interview with a physician, ID28.)

The acceptance was achieved through the presentations (Table 2) of the strategy that aimed at increasing staff awareness of the need for behavioural and environmental changes. On the other hand, the staff who had not participated in the presentation from the beginning of the study had difficulty accepting the project and understanding its scope. By identifying this gap, we realised there was a need for repeating the intervention presentation until all participants had attended the intervention.

### 3.3. Appropriateness

Overall, the staff expressed that the implementation interventions were meaningful and appropriate in making behavioural and environmental changes in their unit.

*“Finally, someone understands us and can see what is really going on. It’s great to have that acknowledgement”.*

(Observation of the intervention presentations for RNs, ID37)

Even the physicians who needed to change their behaviours, for example, by integrating RNs and nursing care in the patient round and at the interdisciplinary meetings, found the implementation strategy valuable.

*“You have my full support in integrating nurses and nursing care in patient rounds. Because I need RNs, their knowledge, and observations of the patients”.*

(Informal individual interview with a physician, ID28.)

This behaviour change enabled RNs to present their observations, evaluations, and plans for nursing care that were taken into consideration by physicians in creating the overall patient treatment plans. The RNs and licenced practical nurses expressed that the implementation interventions such as lectures, group training, bedside training, and individual supervision were helpful in enhancing their awareness and understanding of their professional roles, goals, and responsibilities. These interventions also improved their skills to systematically plan, perform, document, evaluate, and adjust nursing care in collaboration with the interdisciplinary team.

*“When you …started talking about patient care plans, I thought, ‘Oh no not again! It never made sense to me before … but … the way you are training us to develop and apply patient care plans… this is the first time it makes sense to me”.*

(Individual interview, with an RN, ID 27.)

Although registered nurses generally faced difficulty in changing their habits, they were convinced that it was the combination of the implementation interventions that supported them in changing their behaviour and to provide high-quality nursing care delivered according to the patients’ fundamental needs.

*“I really had a hard time figuring out what my professional role was in the beginning of the project. It was like everything was turned upside down, and I was very unsure about what was expected of me. But it makes sense now. It’s so much better. It’s nursing care I need to focus on, and that’s something I can do. Not everything else as I did before. But I would also say that if you had just put some guide in my hand, then I would not use it. I would probably not even read it. But we have really chewed it, we have talked so much about what our professional role, goals, and tasks are. We have been trained in focusing on nursing care on the bedside and at whiteboard meetings. It was a combination of all those interventions, and probably that is what has led to the biggest change”.*

(Focus group interview with RNs, ID46.)

RNs and licenced practical nurses further experienced that behavioural and environmental changes in their unit had provided them with more time for nursing care, thereby indicating that the multifaceted strategy targeted appropriate determinants at the individual, team, management, and environmental levels.

*“I must admit that I have always been in doubt and nervous about whiteboard meetings because I was not sure of what to say. I needed to be prepared and read up on biomedical information that I knew nothing about. But now, now I do not have to be nervous. Now I know what I need to focus on. And that is right… I’m really saving time. I have time to take care of my patients instead of reading medical records. It makes so much more sense”.*

(Focus group interview with RNs, ID30.)

### 3.4. Fidelity

Overall, most of the implementation interventions were performed as intended and in accordance with the original plan. However, there were numerous challenges that affected the fidelity and key persons’ adherence to the strategy, thereby resulting in deviations from the original plan. Eighteen weeks after the beginning of the project, the project was put on hold for 10 weeks due to the COVID-19 pandemic. Consequently, supervision and lectures of evidence-based nursing care regarding nutrition and fluid intervention were delayed or not executed.

Although the implementation process continued after 10 weeks, the COVID-19 pandemic had resulted in an overload of patients, sick leave among the staff, changes in shift plans, and a very busy working schedule for key persons, as they were needed to take on direct patient care. Thus, despite their willingness, the key persons had difficulties integrating the execution of the implementation interventions in their everyday practice. In particular, the execution of bedside training for RNs was experienced as time consuming. Therefore, the researcher (first author) helped to conduct implementation interventions, such as bedside training, individual feedback, and supervisions. Consequently, this inhibited the sustainability of the implementation. The key persons expressed at one point that they lacked ownership for the implementation process. Thereafter, the implementation plan was adjusted by systematically planning and coordinating with the key persons, resulting in a detailed schedule outlining responsibilities, timing, procedures, and coverage for sick leave. The plan was evaluated and revised weekly. Subsequent observations and interviews indicated this change helped to regain ownership of the strategy.

### 3.5. Feasibility

The implementation interventions were, in general, experienced as feasible as they were included in the unit’s routines, without disturbing the time schedule of their clinical practice. Field observations further highlighted that the key persons executed the implementation interventions as intended and supported each other in the execution process, although they found interventions such as supervision and individual feedback challenging and time consuming. Furthermore, the materials supporting implementation interventions were all considered to be transparent, useful, and easy to follow. In particular, the whiteboard magnets in the patients’ rooms, which targeted staff awareness, were experienced as supportive in reminding the RNs to be aware of, and deliver, nursing care in accordance with the patients’ individual needs.

*“It’s so much easier with these magnets. I don’t have to think ahead or remember what the individual patient needs are. It reminds me to focus on interventions that are often overlooked. It helps me remember to brush the patient’s teeth, how much fluid they need, etc. It also saves a lot of my time because I don’t have to go to the computer and look it up. It’s all right here next to the patient, and I can remember everything while I’m here. It also helps the patient remember what they need to do and what the care plan is. I’ve become dependent on them. Physicians are also good at using them, especially for things like oxygen and fluids”.*

(Informal interview with an RN, ID69.)

Although the implementation interventions were feasible, further expansion of bedside training was needed to address staff needs for additional knowledge, understanding, and skills related to their professional roles and tasks. This was the case for RNs facing language barriers and those with low competencies. Expansion or repetition also occurred when the aims or methods were unclear to RNs, thereby leading them to perceive the bedside training as a test rather than support, thereby hindering their learning ability. These challenges, along with project time constraints, impeded the execution of other implementation interventions such as lectures and supervision of nutrition and fluids. Furthermore, halfway into the project, the unit nurse manager was forced to take on the leadership of an extra medical unit. This left little time to implement organisational changes as planned, and cooperation with the hospital kitchen did not succeed as planned.

### 3.6. Penetration

Two-and-a-half months into the project, the nurse manager experienced behavioural changes in the unit.

*“I can see what a difference it makes in our unit. There has been a lot of focus and talk about nursing and performance of nursing care. Nursing care is in demand. Not only by me, but also by the nursing staff and by the physicians”.*

(Informal interview with the nurse manager, ID47.)

Focus group interviews, observation, and audit data illuminated that the staff responded positively to the implementation interventions and adjusted their behaviour according to requests and instructions. The RNS experienced facilitation of nursing care by the management, particularly at whiteboard meetings, having crucially changed their own and their colleagues’ behaviours, such as awareness and social influence. RNs and nursing care became visible and tangible in the interdisciplinary cooperation. The facilitation of nursing care, as well as the facilitation of structured and planned interdisciplinary cooperation, also had a time-saving effect. Instead of RNs reading medical records on biomedical information relevant for physicians, physicians read the records themselves. Moreover, the structured cooperation in a team was experienced to reduce disturbances caused by interprofessional colleagues, which otherwise hindered RNs in delivering nursing care. The saved time was used to provide fundamental nursing care.

*“RN 1: I have got a lot of time to perform nursing care. RN 2: It works ten times better than it did before. The 15 min extra in the morning means the world to me, because I have time for the patients now. I can go out to them right away and begin patient care. I can talk to the patients. RN 1: When I’m out there, I have more focus on the individual patient and on care related to their needs, not everything else like I was doing earlier”.*

(Follow-up (three months) focus group interview with the RNs, ID46.)

Moreover, interdisciplinary cooperation was experienced as being improved.

*“RN 1: Interdisciplinary collaboration has also become much better, because it really requires that we collaborate, and we have control over each of our areas. RN 2: Working with physicians has improved a lot. They really want to hear about nursing, what we observe and assess. I’m allowed to talk about nursing, and I am being listened to. Nursing has become an important part of our collaboration”.*

(Follow-up (three months) focus group interview with the RNs, ID46.)

In general, the RNs expressed, at the end of the project, that the strategy had improved nursing, nursing professionalism, professional identity, and professional terminology in their unit.

*“I thought that I did focus on nursing care, but I have become aware that I did not. I had just focused on patients’ blood tests and what treatment they received, but not nursing care”.*

(Individual interview with the RN, ID27.)

*“Nursing care has reclaimed its value”.*

(Focus group interview with the RNs, ID46.)

Further, patients also experienced an improved quality of care:

*“I do not know what, but something has happened in this unit. I can say that because I’ve been admitted many times before in this unit. Let’s put it this way, it has not been a good experience the other times. I felt abandoned. They just sat there, behind their computers and rarely came across one. But… this time… something has happened in the unit… with the staff. This time, they come all the time and offer help. But they not only ask they also actually do it too. They ask me if I need fluids, food. But not only food as such, they offer it according to my needs. They also ask if I need to be washed and I get help. I really feel that my human needs are being met. My needs are seen and listened to. It’s completely different, and then there is a nice calmness in unit”.*

(Audit, follow-up interview with a patient, ID48.)

**Figure 1:**
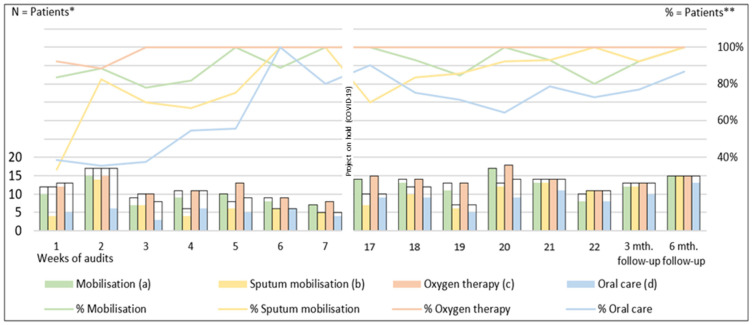
Numbers and proportion of patients who needed and also received the required nursing care interventions regarding their needs (mobilisation, sputum mobilisation, oxygen therapy, and oral care); *—the uncoloured bars represent the total number of patients (admitted ≥24 h) who needed an intervention. The coloured bars illustrate the number of patients who received intervention according to their needs. In total, 195 patients were assessed within 15 audit periods. **—the percentage of the assessed number of patients who received nursing care interventions.

The impact of the strategy was also evident in the audits of electronic patient records. We observed an increase in documentation of performed evidence-based nursing care interventions (Figure 1).

### 3.7. Sustainability

At the three- and six-month follow-ups, the key persons continued to execute the implementation strategy, but not as systematically and focused as earlier. The only implementation intervention that was not maintained was the bedside training, which was due to a lack of time and resources.

*“Yes, it’s not because we do not want to. You must not think that. I would really like to execute the bedside training as it makes so much sense. But it is difficult to find time for it because there is so much else, I also need to do. You have seen my calendar, it is crammed, … then still, something unexpected comes up all the time… We want to, but it is difficult to find the time and resources”.*

(Focus group interview with key persons, ID34.)

However, focus group interviews revealed that RNS who had not received the bedside training requested that they receive it, and the RNs who had received it during the project period wanted more of it. Furthermore, RNs who had received all implementation interventions maintained focus on their professional role, goals, and tasks when cooperating with the team of licenced practical nurses and physicians. The interdisciplinary team continued to support RNs and their provision of nursing care. Lastly, RNs experienced that the strategy had supported them to regain the professional respect they deserved.

At the six-month follow-up, the key persons simplified the bedside training, finding that the original approach too demanding. Rather than addressing individual barriers and tailoring the training accordingly, the focus shifted to improving specific nursing care skills. Additionally, both RNs and licenced practical nurses reported challenges in providing systematic care due to staff shortages and increased workload related to the COVID-19 pandemic.

### 3.8. Costs

The costs of this study consisted mainly of the use of working time (salary cost) for project management as well as planning, execution, evaluation, and adjustment of the interventions. An overview of the time spent by each participant is presented in Table 3. As the materials (pocket cards, whiteboard magnets, etc.) were developed and designed by the first author using the units’ office supplies, the costs for the materials were negligible.

## 4. Discussion

Our findings revealed that the healthcare professionals and key persons perceived the strategy as adaptable, acceptable, appropriate, and feasible. The strategy successfully targeted complex environmental determinants and facilitated behavioural changes, enabling the implementation of fundamental evidence-based nursing care. One of the prerequisites for this success was that the strategy included multiple implementation interventions—such as lectures, presentations, information, and feedback—targeting participants’ understanding and knowledge of the problem and need for change. Importantly, the implementation interventions were tailored to contextual determinants and each profession’s needs and goals, which aligned with the participants’ preferences, fostering motivation and a positive attitude towards the changes in clinical practice. This result is in concurrence with a systematic review by Kwasnicka [32], which reported that the motivation and goal setting that corresponds with participants’ preferences are effective and supportive in changing professional behaviours. Another noteworthy prerequisite for feasibility, also found in other studies [33], was that implementation interventions were seamlessly integrated into the routines of the local unit. Although the content of the implementation interventions was new for the key persons and study participants, its execution did not necessitate additional costs, nor did it disrupt routine practice.

The assessment of the outcome ‘penetration’ also revealed that changing environmental determinants and behaviour at multiple levels led to an improvement not only in the nursing care and interdisciplinary cooperation but also appeared to strengthen nursing professionalism and nurses’ professional identity. A systematic review by Cornett et al. [34] found that professional identity in nursing is influenced by workplace structures, professional hierarchies, the biomedical model, and engagement with evidence-based practises. The review also highlighted that structured training or fellowships strengthen nurses’ professional identity and professionalism, a finding consistent with our study, which emphasises the importance of professional growth opportunities in enhancing both identity and nursing care quality.

The RNs experienced having more time for fundamental nursing care, which was also noticed by the patients. This result was achieved despite our identification of several threats to fidelity. For example, the time constraints and competing organisational demands that were identified as new and unexpected barriers during the intervention period hindered the execution of implementation interventions as planned, which also affected sustainability at three- and six-month follow-ups. These barriers persisted despite the allocation of time and resources. The implementation interventions were initially planned and integrated into existing work patterns to prevent time scarcity and the implementation overload on staff. The scarcity of time has also been identified as a significant barrier to implementation in a systematic review by Geerligs [35], who highlights the importance of awareness of barriers hindering successful implementation during the implementation process. This awareness is particularly crucial when delivering complex, multifaceted interventions, which are often implemented in hospital settings [35]. Therefore, an ongoing process of reflection and evaluation is essential, as well as engagement of implementers (project organisation) in intervention design, frequent involvement, and dialogue with project participants throughout the implementation period [35]. Considering recommendations from [35] and our findings, we emphasise the importance of continual monitoring during the intervention period to identify and address new and unexpected barriers that could threaten the success of implementation. Moreover, based on our findings, we stress the value of ongoing meetings with the project organisation during the intervention period, who by use of monitoring are able to identify and adjust the implementation strategy in a timely manner, aligning it with participants’ preferences and the contextual conditions.

Furthermore, in concurrence with previous research [36,37,38,39], we highlight the crucial importance of involving clinicians, particularly managers who have a thorough understanding and knowledge of the context, in designing the strategy. The acknowledgement of a clinician’s professional opinion and accommodation of their needs as well as those of the environment should be considered when designing and implementing such a strategy. Their involvement, for example, through the co-design process [40], can ensure that the developed multifaceted strategy aligns with the available time and economic resources. Although our study does not illuminate any causality, our findings indicated that the execution of the strategy needs to fit routine clinical practice, and timeframe for implementation of multicomponent strategies should be extended, which is also asserted to improve the desired sustainability [41].

In line with previous research [38,42], one of the most challenging tasks in our study was the achievement of environmental change. Despite managers’ efforts to collaborate with external partners—such as the central kitchen—to implement organisational changes aimed at enhancing RNs’ ability to provide nutritional support, we failed. This result confirms the daily challenges faced by RNS and nursing care staff within a hierarchical and complex healthcare setting [23,43] and indicates the critical need for managers to develop extended implementation skills to successfully drive organisational change. Particularly, as managers are recognised as prime facilitators in the implementation process and possess the power to mediate clinical practice [44,45,46,47]. Considering the consequences of inadequate fundamental evidence-based nursing care and the environmental barriers to its delivery, we argue for a reconceptualization of how nursing care is valued and performed. This requires challenging assumptions about its importance, not only among RNs but also among managers, interdisciplinary professionals, and politicians. Our findings and prior research [43] highlight the need for a paradigm shift or an evidence-based approach that links fundamental nursing care with specialised interventions in a biomedical system, particularly if we want to see improvements in patient safety, care, and cost-effectiveness. Achieving these demands integrating all stakeholders, such as RNs, professionals, management, and policymakers, to drive systemic change.

Although we were unable to achieve organisational change in collaboration with the central kitchen, we successfully targeted other environmental determinants. These included reorganising interdisciplinary cooperation during patient rounds, conducting whiteboard meetings, and eliminating non-nursing tasks assigned to RNs. These changes enhanced RNs’ time and capacity to provide fundamental, evidence-based nursing care and significantly improved interdisciplinary collaboration. This success was largely attributed to the nurse manager, who effectively implemented strategies targeting environmental determinants. This finding underscores the pivotal role nurse managers play in driving successful implementation by leveraging their competencies to address environmental and organisational challenges. Their ability to mitigate environmental determinants demonstrates crucial change management skills and highlights the importance of strong leadership, strategic thinking, and effective communication [44,45,46,47]. Furthermore, these competencies emphasise the need for targeted training and professional development opportunities for nurse managers to further enhance their leadership and implementation capabilities. Empowering nurse managers with these tools is essential for achieving sustainable improvements in clinical practice and fostering a culture of fundamental, evidence-based nursing care.

Considerable knowledge exists on how to design strategies to optimise clinical practice. Previous research reveals that implementation interventions selected and tailored to contextual needs are more effective than no interventions or interventions that are not well tailored to contextual determinants [39,48,49]. Considering these recommendations, our strategy was developed by using prospectively identified determinants in clinical practice, which influenced the delivery of fundamental, evidence-based nursing care. Guided by the intervention mapping framework, the identified determinants were mapped to theory-informed change techniques, thereby resulting in the development of a tailormade multifaceted strategy. Although, the design process has been described as complex and time consuming [24], the findings of this study indicate that the execution of the multifaceted strategy has been an investment for the benefit of RNs and nursing care. In Peters’ scoping review [39], which assessed 118 studies that evaluated implementation interventions, the majority of studies were found to employ multifaceted strategies (75%) with a range of 2–13 implementation interventions.

However, only a few studies have developed strategies that target change at multiple levels [18,50]. The focus lies mostly on individual or organisational determinants. Considering that nursing practice and decision-making typically takes place within a team, RNs’ actions and decisions may consequently be influenced by various factors such as other professions, working culture, management, policies, and resources. This was also the case in our previous study [23], thereby indicating the need for the development of a strategy that targeted not only RNs and their delivery of nursing care but also the entire working environment. Moreover, as stated in a systematic review by Cassidy [50], for RNs to be able to deliver high-quality care, there is a need to deviate from the assumption that insufficient nursing care is caused by mono-professional incompetence. Moreover, a recent systematic review by Boaz [51] on strategies for translating research evidence into practice emphasised the importance of assessing both mono-professional and contextual determinants that may affect nursing care. This assessment should be used to tailor multifaceted implementation interventions targeting determinants at multiple levels (individual, team, management, and organisation). Consequently, implementation science recommends the use of tailored multifaceted strategies comprising research- and theory-based implementation interventions, as this leads to potentially stronger effects than single intervention strategies (e.g., educational strategies) [33,50,51]. In contrast, the previously mentioned scoping review by Peters [39] found that despite a majority of studies (82%) achieving improvements in one or more reported outcomes (most often in professional behaviour), the beneficial outcomes were achieved regardless of whether the strategy was single or multifaceted. Thus, future research is warranted in order to establish whether tailored multifaceted interventions targeting contextual determinants are associated with impact.

### Strengths and Limitations

To evaluate the implementation strategy, we applied the implementation outcomes framework and primarily utilised qualitative data to structure the evaluation process. Utilising this framework provided a significant advantage, as it enabled us to assess implementation process outcomes considered the ‘gold standard’ in implementation research [52]. This approach enhanced our understanding of the implementation process, participants’ experiences, and contextual influences. Additionally, a systematic review by Boaz provides evidence that qualitative approaches offer detailed insight into implementation interventions and their implementation [51]. However, when evaluating the feasibility of a multifaceted strategy, quantitative methods that test the effect of the strategy on patient outcomes would have been valuable for understanding the impact of a multifaceted strategy on nursing practice. It is important to note that when considering our result regarding ‘penetration’, the results must be interpreted with caution, as the first author was deeply involved in the implementation process by helping implementers to execute the implementation interventions as well as collecting data. It is possible that the involvement of the researcher constituted an unnatural setting for implementation. Moreover, it may have been a threat to sustainability, as the key persons possibly needed to be more involved in the implementation process than planned. Hence, for future studies that would aim to address sustainability challenges, it is imperative to develop strategies tailored to organisational capacity. Accordingly, incorporating approaches that empower implementers to enhance their implementation competencies will be vital for effectively executing multifaceted strategies. Furthermore, our study period lasted only eight months and that is possibly too brief to assess penetration and sustainability. Therefore, future studies should consider a longer intervention and follow-up period, particularly when applying complex multifaceted strategies.

Furthermore, the strategy in this study was tailored to the local context and is, therefore, not transferrable to another context. However, our results indicate that the method (intervention mapping framework) used to develop our strategy and the systematic implementation process is transferrable.

## 5. Conclusions

This study assessed whether a tailored, multifaceted implementation strategy was feasible for changing healthcare professionals’ behaviour and environmental determinants that hinder RNs from delivering fundamental evidence-based nursing care in accordance with patients’ needs. By utilising the implementation outcomes framework for determining the implementation process outcomes, we identified that the strategy successfully addressed and targeted complex environmental determinants and facilitated behavioural changes at the individual, interdisciplinary team, and management levels. Despite the identified challenges regarding fidelity, the implementation strategy interventions penetrated clinical practice and targeted the delivery of fundamental evidence-based nursing care, increased interdisciplinary cooperation, and involved the reorganisation of working processes. However, the study period was brief and influenced by competing demands from the organisation, thereby limiting the sustainability at the three-and six-month follow-ups. To fully understand whether the strategy could successfully be employed in other hospital units and for another group of patients, further research is warranted to test the strategy in a larger intervention study. Furthermore, further research is needed to investigate the effect of the strategy on nursing-sensitive patient outcomes as well as health–economic outcomes.

## Figures and Tables

**Table 1:** Definition of implementation outcomes and the overview of data collection methods.

Implementation Outcomes [Proctor 2011]	Definition *	Data Collection
Adoption	The intention, initial decision, or action to try to employ an innovation or evidence-based practice	Field observations
Acceptability	The perception among project managers, implementers, and key persons that a given treatment, service, practice, or innovation is agreeable, palatable, or satisfactory	Field observations
Appropriateness	The perceived fit, relevance, or compatibility of the innovation or evidence-based practice for a given practice setting, provider, or consumer, and/or perceived fit of the innovation to address a particular issue or problem	Field observations Audit of electronic patient records
Fidelity	The degree to which an intervention was implemented as prescribed in the original protocol or as intended by the programme developers	Field observations Registration
Feasibility	The extent to which a new treatment, or an innovation, can be successfully used or carried out within a given agency or setting	Field observations
Penetration	The integration of a practice within a service setting and its subsystems	Field observations Audit of electronic patient records Focus group interviews
Sustainability	The extent to which a newly implemented treatment is maintained or institutionalised within a service setting’s ongoing stable operations	Field observations Audit of electronic patient records Focus group interviews
Costs	The cost impact of an implementation effort	Registration

* Description of the outcomes is adapted from Proctor et al. [26].

**Table 2:** Overview of the multifaceted implementation strategy describing targeted behaviours and determinants, delivery mode, materials, and dose.

Targeted Behaviours and Environmental Determinants	Implementation Interventions	Intervention Content	Delivered to	Mode of Delivery and Materials	Dose
Knowledge Beliefs about consequences	Presentation	Provide information and stimulate meaning of the following: Previous research results of missing and unsystematic performance of nursing care and its consequences [27]; Barriers and facilitators for delivering fundamental evidence-based nursing care [23]; Execution and content of the implementation strategy.	RNs Interdisciplinary team (all healthcare professions and management)	Face-to-face (group) Open debate Folders of the project Newsletters	Mono-professional group sessions, 2 × 30 min
	Information	Progress of the implementation strategy	RNs Interdisciplinary team	Face-to-face (group) Newsletter	1 × weekly 15 min 1 x weekly
	Lectures	Provide knowledge and stimulate meaning of the following: Professional role, tasks, goals, and terminology; Assessment of patient individual needs; Systematic planning, execution, and documentation of fundamental evidence-based nursing care (nutritional support, fluid therapy, oral care, ambulation, oxygen therapy, and sputum mobilisation); The impact of person-centred care: integration of patients in care plan; Development and use of the care plan; Systematic cooperation with the interdisciplinary team with a focus on integration of fundamental evidence-based nursing care.	RNs Interdisciplinary team	Face-to-face (group) Educational materials (videos, PowerPoint, quiz, etc.) Clinical pathway *	Daily, 15 min sessions and 2 × 45 min group sessions.
Skills	Bedside training	Individual training followed by feedback in the following: Systematic assessment, performance, evaluation, adjustment, and documentation of fundamental evidence-based nursing care according to patient individual needs; Cooperation with the interdisciplinary team according to own professional role, goals, and tasks.	RNs	Face-to-face (individual and in group) Training guide Evaluation sheets Learning contract for goal setting Clinical pathway *	8 h session per person
	Supervision	Provide guidance and supervision based on individual needs to enhance skills in delivering fundamental evidence-based nursing care, identified through weekly monitoring	RNs	Face-to-face (individual)	Ad hoc
	Group training	Group training to increasing skills in the following: Documentation, development, and use of patient care plans; Use of products to provide fundamental evidence-based nursing care (e.g., oxygen therapy and oral care products); Presentation of relevant nursing observations and evaluation of patient status at interdisciplinary cooperation meetings and at patient rounds.	RNs (group)	Face-to-face (group) Educational materials (videos, PowerPoint, quiz, etc.) Clinical pathway describing in detail evidence-based criteria for delivery of fundamental nursing care initiatives.	Daily, 15 min sessions. Executed in combination with lectures
Memory, attention, and decision processes; social influence; and beliefs about capabilities	Reminders, facilitation/ elimination	Promote RNs attention, memory, and consciousness by reminding, facilitating, and encouraging the following: Performance of fundamental evidence-based nursing care; Nursing roles, tasks, and goals; Professional terminology; Elimination of non-supportive implementation behaviours; Interdisciplinary cooperation; Shifting focus from biomedical and administrative tasks to address individual patient needs for fundamental evidence-based nursing care; Using professional terminology during interdisciplinary collaboration and when documenting care plans. Eliminate hierarchical dominance and social influence by performing the following: Facilitation and encouragement of RNs to collaborate with the interdisciplinary team with focus on nursing care and not only on biomedical and administrative tasks; Addressing and eliminating non-supportive behaviours within the interdisciplinary team, e.g., delegating non-nursing tasks to RNs.	RNs Interdisciplinary team	Face-to-face (individual and in group) Prompts and cues Whiteboard magnets Pocket cards Guidance of facilitating/eliminating strategies	Individually (ad hoc). In groups at daily mono-, professional-, and at interdisciplinary whiteboard meetings
	Feedback	Facilitate high involvement and increase motivation by giving feedback on implementation progress and results of individual and collective performance: Feedback is given based on results from the continuous monitoring. Discussion and reflection are encouraged when giving feedback.	RNs Interdisciplinary team Management	Face-to-face (individually and in group)	Individual feedback (ad hoc) Feedback of the implementation progress, monthly, in group
	Nudging	Prompting materials increasing memory, attention, and decision process: Whiteboard magnets on patient whiteboards prompting memory to provide fundamental nursing care in line with evidence-based recommendations and patient individual needs; Pocket sheets with daily overview of admitted patients and their needs for fundamental evidence-based nursing care; Pocket cards reminding each profession’s roles, tasks, and goals during collaboration activities (e.g., at patient rounds and at whiteboard meetings).	RNs Interdisciplinary team		Daily use
Environmental determinants	Environmental changes	Reorganisation of working procedures, increasing RNs time and ability to provide fundamental evidence-based nursing care: Eliminate tasks delegated to RNs by other professions (e.g., bringing or ordering food for patients from the central kitchen instead of the kitchen staff, ordering blood tests for physicians instead of physicians, cleaning tasks, etc.); Reorganise interdisciplinary cooperation at patient rounds; Reorganise interdisciplinary cooperation at whiteboard meetings to increase time to provide fundamental nursing care; Reorganise cooperation with the central kitchen to increase RNs ability to provide nutrition therapy.	RNs Interdisciplinary team Management		Daily cooperation

* Clinical pathway included description of evidence-based criteria for the delivery of fundamental nursing care interventions. Abbreviation: Registered nurses: RNs.

**Table 3:** Overview of estimated time spent executing the strategy during the eight-month period.

Project Organisation	Time Spent (Hours Per Month)	Intervention (For Further Clarification, See Table 2)
First author	160	Project management, planning, adjustment, and execution of the implementation interventions and data collection
Second and third author	10	Guidance and supervision
Last author	20	Guidance and supervision and participation in data analyses
Head nurse	3 (total)	Adoption and facilitation of the project
Nurse manager	16	Planning, adjustment, execution, and maintenance of the implementation interventions
Nurse manager assistants (*n* = 2)	16	Execution and maintenance of the implementation interventions
Clinical nurse specialist	80	Planning, adjustment, execution, and maintenance of the implementation interventions, as well as data collection
Registered nurses (*n* = 3)	8	Support and maintenance of evidence-based nursing care

## Data Availability

Data are contained within the article or Supplementary Materials.

## Supplementary Materials

The following supporting information can be downloaded at the following website: https://www.mdpi.com/article/10.3390/nursrep14040297/s1, Table S1: An overview of evidence-based guidelines criteria for nursing care interventions for older patients admitted at medical unit. References [53,54,55,56,57,58,59] are cited in the Supplementary Materials.

## Public Involvement Statement

There was no public involvement in any aspect of this research.

## Guidelines and Standards Statement

This manuscript was drafted according to the Standards for Reporting Implementation Studies (StaRI) checklist for implementation research.
