# Supplementary material for: Tailored Multifaceted Strategy for Implementing Fundamental Evidence-Based Nursing Care: An Evaluation Study
Source: Nurs Rep. 2024 Dec 18;14(4):4070–90. doi: 10.3390/nursrep14040297 (PMC11677046; doi:10.3390/nursrep14040297)
Supplement: Supplementary file 1 [file nursrep-14-00297-s001.zip › nursrep-3244735-supplementary.pdf]

Supplementary material, Table S1. An overview of evidence-based guidelines criteria for nursing care interventions for older patients admitted at medical unit.

Indicator for nursing care interventions	Recommended intervention	Level of evidence*	Recommending EBG
**Sputum mobilisation**	Airway clearance techniques by Positive Expiratory Pressure when patients have difficulty with expectoration or in the event of a pre-existing lung condition.	D	[53,54]
**Oral care**	Tooth brushing minimum twice a day (minimum 2 minutes) with toothpaste containing 1000-1500ppm fluoride.	B	[55]
**Fluid therapy**	Assess volume depletion and fluid status daily. Develop target fluid therapy plan when discrepancy between fluids intake and output, electrolyte disturbances, abnormal pulse and blood pressure and in presence of: confusion, diarrhoea, vomiting. Advise to drink plenty of fluids.	C	[53,54,56,57]
**Nutritional support**	Screen nutrition status within ≤24 hours of admission and develop targeted nutrition support plan for patients at risk of malnutrition (assessed by: BMI (<20,5), weight loss ≤3 months, reduced dietary intake ≤1week, age (>70 years) and health condition (chronic illness, bedridden, in intensive care).	C	[53,54,56,57]
**Mobilisation**	Assess functional ability and develop targeted mobilisation plan for patients with loss of functional ability (in conjunction with hospitalisation), who need mobilisation support (for activities of daily living) or rehabilitation. Mobilize patients (walk or sit out of bed) within ≤24 hours of hospitalisation, for 20 minutes, and increase mobilisation each subsequent day.	A	[53,54,58]
**Oxygen therapy**	Oxygen therapy must be guided by the level of arterial oxygen tension (PaO_2) and oxygen saturation (SpO_2). For acutely ill patients (not at risk of hypercapnic respiratory failure) PaO_2 is >8 kPa and SpO_2 94–98%. For patients with COPD or who are at risk of hypercapnic respiratory failure, SpO_2 is 88-92%.	D	[53,54,59]

* The level of evidence is assessed by referred evidence-based guidelines.

References

53. Danish Society of Respiratory Medicine. Guidelines for pneumonia - initial assessment and treatment. 2010 (revised 2021). Available online: https://lungemedicin.dk/wp-content/uploads/2022/01/DLS_DSI_Pneumoni_2021_110122_CPF.pdf (accessed on 1 July 2019).
54. Lim WS, Baudouin SV, George RC, Hill AT, Jamieson C, Le Jeune I et al. BTS guidelines for the management of community acquired pneumonia in adults: update 2009. Thorax. 2009; 64 Suppl 3:iii1-55.
55. Frølund JC, Klit MØ, Ladegaard L, Lytjohan M, Mielcke H, Nielsen BE et al. Clinical Guideline- Oral hygiene for adult citizens and patients 2016. http://cfkr.dk/media/351666/Mundhygiejne%20til%20voksne%20borgere%20og%20patienter.pdf. (assessed 1 July 2019).
56. The Danish Diet & Nutrition Association. The National Nutrition Handbook. 2016.Available online: https://xn--kosthndbogen-xcb.dk/ (accessed on 1 July 2019).
57. The Danish Health Authority. Knowledge base: Nutritional interventions for the older medical patient. 2017. Available online: https://www.sst.dk/da/udgivelser/2017/~/media/3AD0A0709E57494EB8726B9D004CAE00.ashx. (assessed 1 April 2019).
58. Woodhead M, Blasi F, Ewig S, Garau J, Huchon G, Ieven M et al. Guidelines for the management of adult lower respiratory tract infections--full version. Clin Microbiol Infect. 2011;17 Suppl 6:E1-59.
59. O'Driscoll BR, Howard LS, Earis J, Mak V. British Thoracic Society Guideline for oxygen use in adults in healthcare and emergency settings. BMJ open respiratory research. 2017; 4(1):e000170.
